# Radiation therapy vault shielding calculational methods when IMRT and TBI procedures contribute

**DOI:** 10.1120/jacmp.v2i3.2609

**Published:** 2001-09-01

**Authors:** James E. Rodgers

**Affiliations:** ^1^ Department of Radiation Medicine Georgetown University Hospital 3800 Reservoir Road NW Washington, D.C. 20007

**Keywords:** shielding, workload, use factor, IMRT, TBI, electron accelerators

## Abstract

The additional intensity modulated radiation therapy (IMRT) and total body irradiation (TBI) to conventional treatment clinical treatment procedures can significantly increase the contribution of accelerator head leakage radiation. Previously recommended procedures by the National Council on Radiation Protection and Measurements (NCRP) for vault design, specifically calculations of primary and secondary barrier thicknesses, are not valid when leakage radiation significantly exceeds direct radiation. Use factor distributions are also influenced by IMRT and TBI procedures. Methods are proposed to extend the NCRP barrier design formulas to resolve these problems. The medical accelerator (weekly) workload is separately determined for the direct, leakage, and scatter radiation components. Applications of the formulas to the calculation of primary and secondary barriers are discussed. The addition of IMRT to the shielding design is explored as a function of the fraction patients receiving IMRT and the MU to dose ratio. Secondary barrier thicknesses could be increased by as much as 1 TVL.

PACS number(s): 87.52.–g, 87.53.–j

## INTRODUCTION

In the last 15 years, the technology of treatment delivery for radiation oncology patients has undergone tremendous change. In particular, multileaf collimator based intensity modulated radiation therapy (IMRT), in its various versions, has been found relatively inefficient compared to conventional treatment methods. The nature of the inefficiency is that more accelerator monitor units (MU) are required per unit dose to the target volume. In brief, the reason is that only a fraction, often small, of each treatment field is being irradiated at any instance of delivery. The ratio, *C*, of the number of MU to dose (number of cGy) at the isocenter has been found to range^1^ ^–^ ^5^ from 2 to 10, with some proposed methods going even higher.^6^ ^–^ ^7^ This increased MU load means the leakage radiation from the accelerator assembly has increased by a factor of approximately *C* and raises concern about the adequacy of radiation protection afforded by existing facilities now embarking on IMRT as well as how to design new shielded vaults for IMRT.

Another treatment technique, total body irradiation (TBI), also significantly raises the number of MU needed to deliver the prescribed dose at the patient. TBI is performed with the patient at extended distances from the isocenter (typically 2 to 5 m) and therefore requires 9 to 36 times more MU than if the patient were at the isocenter. TBI, however, contributes no scatter from the isocenter while producing a higher direct radiation incident on the primary barrier behind the patient.^8^

There are three principal sources of ionizing radiation incident on protective barriers: direct, leakage, and scatter. These radiations have, in general, different penetrating qualities and tenth value layers (TVL). The NCRP Report No. 49 method for primary barrier thickness calculation to attenuate direct radiation starts with the formula:^9^

$$P=B\;\;\;WUT/d^{2}.$$

Here *P* is the design dose equivalent limit (some fraction of 1/50 of the regulatory annual limit) for the site, *S*, being protected; *B* is the barrier transmission factor. *W* is the radiation workload in terms of total dose delivered at the isocenter (1 m from x‐ray target) per average week. *U* and *T* are the use and occupancy factors, respectively. *d* is the distance from target to the point *S*. Similar formulas for determining secondary barrier thickness requirements stemming from the leakage and patient scatter are also proportional to the workload *W*. One commonly used procedure for estimating *W* is to divide the sum of all patient treatment prescribed doses at isocenter per week by a representative tissue‐maximum‐ratio or equivalent. The implicit assumption for leakage calculations is that 1 cGy of dose at isocenter requires approximately 1 MU (i.e., C≈1). IMRT procedures violate this assumption because the ratio of total MU to dose delivered in regional around the isocenter is significantly larger than 1.

## METHODS AND ANALYSIS

In order to handle the problem, increased leakage radiation produced by IMRT and TBI treatments the calculational will use three types of workloads–one for direct, one for leakage, and one for scatter radiation, respectively. As will be shown below, when the TBI contribution is negligible, only the direct and leakage workloads are needed, since the direct and scatter workloads are equal.

Furthermore, clinical electron accelerators are often multimodal having a low‐energy (LX) and a high‐energy (HX) x‐ray beam and an assortment of electron beams. Each x‐ray beam quality will have separate workloads. If certain criteria are met, the room shielding design calculations may need to be performed only for the HX beam.

The fraction of a workload that the gantry or beam is oriented in a specific direction is the use factor for that direction. As will be illustrated below, when TBI procedures are considered, the direct and leakage use factor sets may be equal but different from the scatter set of use factors. Conversely, without TBI these use‐factor differences are not significant.

### Direct workload $\left(W_{\text{dir}}\right)$ and use factors

The direct workload, $W_{\text{dir}}(\text{QX})$, is the sum of contributions from procedures or activities, which produce direct incidence of radiation of quality QX (LX or HX) on some primary barrier. Direct workloads are specified in units of dose or dose equivalent at 1 m from the target (normally at the isocenter) per week. The direct workload receives contributions from several activities: (a) $W_{\text{conv}}$, conventional treatment delivery, including AP/PA, rotations, 3D‐CRT, and others; (b) $W_{\text{QA}}$, quality assurance activities, which may include annual calibrations and other physics research activities. These workload contributions apply to the extent that they are performed during standard working hours; (c) $W_{\text{SRP}}$, stereotactic radiosurgery and stereotactic radiotherapy procedures; (d) $W_{\text{TBI}}$, total body irradiation dose contribution at the isocenter (1 m from target); (e) $W_{\text{IMRT}}$, intensity modulated radiation therapy, where the ratio of accelerator MU generated to the dose, in cGy, delivered at the isocenter can be much larger than 1 (MU/cGy). Thus, compared to conventional treatment for the same prescribed doses, the leakage radiation is significantly increased.

Each $W_{\text{dir}}$ contribution is in units of dose at the isocenter per week.

For the direct contributions described the total direct workload is

$$\begin{array}{lll} W_{\text{dir}}(\text{QX}) & = & W_{\text{conv}}(\text{QX})+W_{\text{QA}}(\text{QX})+W_{\text{SRP}}(\text{QX})+W_{\text{TBI}}(\text{QX})+W_{\text{IMRT}}(\text{QX})\\ & \equiv & W_{\text{standard}}(\text{QX})+W_{\text{TBI}}(\text{QX})+W_{\text{IMRT}}(\text{QX}). \end{array}$$

Here $W_{\text{standard}}$ is the sum of those direct contributions (other than TBI) having similar ratios of rates of production of primary and leakage radiations.

**Figure 1:**
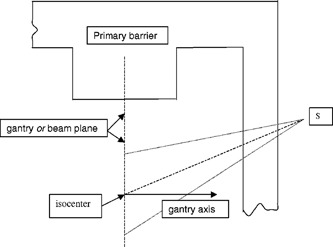
Leakage radiation from the accelerator head to point *S* outside secondary barrier.

**Figure 2:**
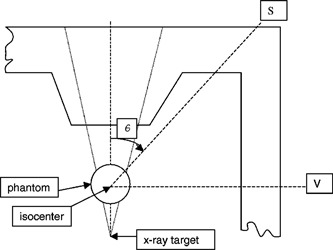
Scatter from patient or phantom located near isocenter.

A *use factor, U*, for a particular gantry orientation, *G*, is the fraction of a workload that the gantry is oriented at angle *G* (or an angular interval with mean value *G*). The use factors for the direct workload may be different from those applicable to leakage or scatter workloads, as illustrated in Figs. 1 and 2.

For the direct workload a use factor applying to gantry orientation $G_{k}$ is denoted $U_{\text{dir}}(\text{QX},G_{k})$. The sum of all use factors $\left\{U_{\text{dir}}(\text{QX},G_{k})\right\}$ should be equal to 1. The contribution of TBI procedures to $W_{\text{dir}}$ can significantly increase the direct use factor for the designated treatment wall orientation.

### Leakage workload $W_{L}(\text{QX})$ and use factors

The secondary barrier workload for leakage arising from x‐rays of primary quality $\text{QX},W_{L}(\text{QX})$, is determined from the total monitor units rendered per week. $W_{L}(\text{QX})$ is converted to dose (DE) at 1 m from the x‐ray target by multiplying two factors: (1) a conversion factor (normally taken to be 1 cGy/MU for $W_{\text{standard}}$), and (2) the leakage attenuation factor specified by the accelerator manufacturer or regulatory agency (FDA). The leakage attenuation factor, $L_{o}$, is by regulation not to exceed 0.1% of $W_{L}(\text{QX})$.

The treatment delivery technique known as intensity modulated radiation therapy, IMRT, contributes more to $W_{L}(\text{QX})$ per unit dose at the isocenter than to $W_{\text{dir}}(\text{QX})$ by a factor *C*. As mentioned above, *C* depends on the specific IMRT technique and equipment used to deliver IMRT and can vary over a range from 2 to over 10. The total leakage workload, $W_{L}(\text{QX})$, is thus given by:

$$W_{L}(\text{QX})=W_{\text{standard}}(\text{QX})+W_{\text{TBI}}(\text{QX})+C^{*}W_{\text{IMRT}}(\text{QX}).$$

Obviously, if C=1 or $W_{\text{imrt}}=0$, then $W_{L}=W_{\text{dir}}$.

*Leakage use factors.* The use factor for leakage when the gantry has orientation, $G_{k}$, is $U_{L}(\text{QX},G_{k})$ and equal to the fraction of $W_{L}(\text{QX})$ that the gantry is oriented in that direction. Particular attention should be given to the gantry orientations for TBI procedures, which can significantly alter the $U_{L}(\text{QX},G_{k})$ distribution.

### Scatter workload and use factors

The scatter workload is determined by the dose at the isocenter received by the patient or phantom. $W_{\text{sca}}(\text{QX})$ receives contributions from $W_{\text{standard}}(\text{QX})$ and $W_{\text{IMRT}}(\text{QX})$, but negligible room scatter arises from $W_{\text{TBI}}(\text{QX})$. Therefore,

$$W_{\text{sca}}(\text{QX})=W_{\text{standard}}(\text{QX})+W_{\text{IMRT}}(\text{QX})⩽W_{\text{dir}}(\text{QX}).$$

Therefore, a conservative and simplifying procedure would be to set $W_{\text{sca}}(\text{QX})\approx W_{\text{dir}}(\text{QX})$ (which is exact when $W_{\text{TBI}}(\text{QX})=0)$.

*Scatter use factors.* The use factor for scatter when the gantry has orientation, $G_{k}$, is $U_{\text{sca}}(\text{QX},G_{k})$ which is the fraction of $W_{\text{sca}}(\text{QX})$ that the gantry has angle $G_{k}$. If $W_{\text{TBI}}(\text{QX})=0$, then $U_{\text{sca}}(\text{QX},G_{k})=U_{\text{dir}}(\text{QX},G_{k})$.

### Combining thickness requirements

The NCRP method for determining the required secondary barrier thickness starts with independent calculations of the thickness needed for scatter and for leakage using a common *P* value. For primary barriers, two independent calculations may need to be performed for the LX and HX modes. The procedure for combining results of barrier thicknesses, $x_{1}$ and $x_{2}$, arising from two sources of ionizing radiation is as follows. (1) If the difference $\left|x_{1}-x_{2}\right|$ is less than 1 TVL of the more penetrating source, then 1 HVL (or 0.31 TVL) of the more penetrating radiation *shall* be added to the larger of $x_{1}$ or $x_{2}$. (2) If source 2 is the more penetrating source and (a) $x_{1}\geq x_{2}+1\;{\text{TVL}}_{2}$, then use $x_{1}$ for the barrier thickness, or (b) $x_{2}\geq x_{1}+1\;{\text{TVL}}_{1}$, then use $x_{1}$, then use $x_{2}$ for the barrier thickness.

### Procedure for use of TVL's

When the first TVL, ${\text{TVL}}_{1}$ is different from subsequent (“equilibrium”) TVL's, ${\text{TVL}}_{\text{eq}}$, the barrier thickness corresponding to a requirement of *n* TVL's is:

$$x={\text{TVL}}_{1}+(n-1){\text{TVL}}_{\text{eq}}.$$

If one were to use $x=n^{*}{\text{TVL}}_{\text{eq}}$ instead, it would result in a conservative thickness since the equilibrium TVL is larger than the first TVL for polyenergetic beams.

## RESULTS

### Primary barrier thickness

The barrier thickness required to achieve a weekly permitted dose equivalent, *P*, at a point *A* beyond a primary barrier irradiated by gantry angle, $G_{A}$, is determined from

$$P=W_{\text{dir}}(\text{QW})*U_{\text{dir}}(\text{QX},G_{A})*T_{A}*10^{-x/\text{TVL}}{\left(1\;m/d_{p}(A)\right)}^{2},$$

where $T_{A}$ is the occupancy factor for point A and $d_{p}(A)$ is the distance along the primary beam central axis from the x‐ray target to point *A*. The factor $10^{-x/\text{TVL}}=B$ the primary barrier transmission factor.

This yields the number of TVL's as:

$$n\equiv x/\text{TVL}=\log_{10}[W_{\text{dir}}(\text{QX})*U_{\text{dir}}(\text{QX},G_{A})*T_{A}/(d_{p}{\left(A\right)}^{2}*P)].$$

For a dual x‐ray mode unit both the high (HX) and low (LX) energy x‐ray barrier thicknesses should be examined. Often the HX quality x‐rays shielding requirements will be adequate for both modes combined.

### Secondary barriers for leakage

(a) Consider a point *S* beyond a secondary barrier as illustrated in Fig. 1. For each gantry orientation $G_{k}$ the leakage contribution to *S* in the absence of a barrier is

$$D(L,G_{k})=W_{L}(\text{QX})*U_{L}(QX,G_{k})*L_{o}*{\left(1\;\;m/d_{L}(S,G_{k})\right)}^{2},$$

where $L_{o}$ is the leakage attenuation factor and $d_{L}(S,G_{k})$ is the distance from the x‐ray leakage source (approximated by the x‐ray target) to *S*. If a barrier is posed between the gantry and *S* for all gantry orientations, the total dose at *S* is found by summing over all gantry orientation contributions. Thus, the number of TVL's required to achieve a weekly limit *P* for a leakage radiation quality QX, is

$$P=W_{L}(\text{QX})*L_{o}*10^{-x/\text{TVL}}*T_{S}*\Sigma_{k}[U_{L}(\text{QX},G_{k})*{\left(1\;\;m/d_{L}(S,G_{k})\right)}^{2}].$$

So,

$$n_{L}(\text{QX},S)\equiv x/\text{TVL}=\log_{10}\{W_{L}(\text{QX})*T_{S}*L_{o}*\Sigma_{k}[U_{L}(\text{QX},G_{k})/d_{L}{\left(S,G_{k}\right)}^{2}]/P\}.$$

(b) A simplification of the above formula occurs when the region to be shielded lies near the gantry rotational axis (as illustrated by point *V* in Fig. 2). In this situation the distance, $d_{L}(V)=d_{L}(V,G_{k})$, from the x‐ray target to point *V* near the rotational axis is approximately the same for any gantry orientation, $G_{k}$. Thus,

$$P=W_{L}(\text{QX})*L_{o}*10^{-x/\text{TVL}}*T_{V}{\left(1\;\;m/d_{L}(V)\right)}^{2}$$

and

$$n_{L}(\text{QX},V)\equiv x/\text{TVL}=\log_{10}\{W_{L}(\text{QX})*T_{V}*L_{o}/[d_{L}{\left(V\right)}^{2}*P]\}.$$

(c) After the number of leakage TVL's required has been determined for the region *S*, the barrier thickness for leakage arising from QX x‐rays is computed by

$$x(\text{QX},S)={\text{TVL}}_{1}(L,\text{QX})+(n_{L}(\text{QX},S)-1)*{\text{TVL}}_{\text{eq}}(L,\text{QX}).$$

### Secondary barriers for scatter and reflection

(a) Part of the radiation dose at a point of interest *S* behind the secondary barrier in Fig. 2 is produced by the scatter of primary beam photons incident on a patient or phantom located at the isocenter. For the illustrated gantry orientation, *G*, and scatter angle, *θ*, the fraction of the primary beam (quality QX) dose at the isocenter which is scattered toward *S* is given by the quantity

*a*(QX, *θ*). The scatter fraction, *a*(QX, *θ*), is the ratio of the scattered radiation dose (to a mini‐phantom of tissue equivalent medium under full buildup at 1 m from isocenter) to the primary beam dose at the isocenter in the presence of the patient (or phantom). Furthermore, *a* is defined for a field size at isocenter of 400 cm^2^ representing an average field size for typical clinical applications. Scattering degrades the quality, QX, of the primary beam and the TVL of the scatter is strongly dependent on the scattering angle, *θ*.^9^ ^,^ ^10^

For the particular gantry orientation illustrated, the dose transmitted to *S* by the secondary barrier is given by

$$D(S,\theta )=W_{\text{sca}}(\text{QX})*U_{\text{sca}}(\text{QX},G)*a(\text{QX},)*(F/400)*{\left(1\;\;m/d_{I}(S)\right)}^{2}*10^{-x/\text{TVL}(\text{sca},\text{QX},\theta )}$$

where *F* is the area of the primary beam field at isocenter, in cm^2^, and *x* is the oblique pathlength of the rayline to *S* in the barrier. $d_{I}(S)$ is the distance from isocenter to *S*, which happens to be independent of gantry orientation. If it was determined that the dominant contribution of scatter dose to *S* came from a single gantry orientation, then the required number of TVL's needed to reduce scatter dose at *S* to a level of *P*, is given by

$$n_{\text{sca}}(\text{QX},S,\theta )\equiv x/\text{TVL}=\log_{10}[D(S,\theta )*T_{S}/P].$$

Therefore, the oblique barrier pathlength, *x*(QX,*S*), is:

$$x(\text{QX},S)={\text{TVL}}_{1}(\text{sca},\text{QX},\theta )+(n_{\text{sca}}(\text{QX},S,\theta )-1)*{\text{TVL}}_{\text{eq}}(\text{sca},\text{QX},\theta ).$$

(b) In general, the fraction and quality (i.e., TVL) of scattered x‐rays can vary significantly with *θ*. Thus, the summation of scatter dose contributions at the point of interest illustrated by *S* in Fig. 2 is of little practical value in determining a barrier thickness for scatter since the TVL's will differ significantly for various gantry (hence scatter) angles. Such a sum of contributions would be useful in evaluation of the total scatter dose transmitted to *S* once the barrier thickness has been determined.

In the special case of scatter in the direction of the gantry rotational axis where θ=90° as illustrated by point *V* in Fig. 2, the sum

$$\begin{array}{lll} D(V) & = & \Sigma_{k}D(V,\theta_{k}=90{}^{\circ})\\ & = & W_{\text{sca}}^{*}(F/400)*{\left(1\;\;m/d_{I}(V)\right)}^{2}*\Sigma_{k}[U_{\text{sca}}(\text{QX},G_{k})*a(\text{QX},\theta_{k}=90{}^{\circ})*10^{-x/\text{TVL}(\text{sca},\text{QX},\theta_{k}})] \end{array}$$

simplifies to

$$D(V)=W_{\text{sca}}(\text{QX})*(F/400)*{\left(1\;\;m/d_{I}(V)\right)}^{2}*a(\text{QX},90{}^{\circ})*10^{-x/\text{TVL}(\text{sca},\text{QX},90)}.$$

**Figure 3:**
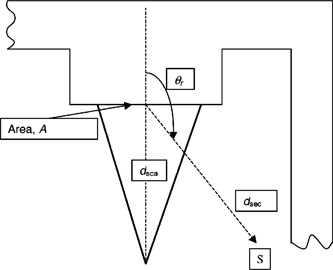
Reflection of direct beam from a barrier surface.

(c) When the source of scatter is not a patient but a barrier located at a distance $d_{\text{sca}}$ from the x‐ray target along the primary beam central axis, then the scatter dose or “*reflection*“ dose from the barrier into the direction indicated by $\theta_{r}$ in Fig. 3 is given by

$$D(\theta_{r},G_{k})=W_{\text{dir}}(\text{QX})*U_{\text{dir}}(\text{QX},G_{k})*(1\;\;m^{2}/d_{\text{sca}}^{2})*(\alpha *A/d_{\text{sec}}^{2}).$$

Here the reflection coefficient *α* for a particular barrier material depends on the quality and angle of incidence of the beam intercepting the barrier. *α* is a function of the reflection or scatter angle. *A* is the barrier area (in $m^{2}$) intercepted by the beam. Wall scatter or reflection often produces scatter radiation at the room door located behind the maze barrier.

## DISCUSSION

The influence of TBI and/or IMRT procedures on the different workloads and use factors will be illustrated by some typical examples:

(a) First, we look at clinical procedures that consist of both “standard” and IMRT treatments but no TBI. Let *r* be the fraction of $W_{\text{dir}}(\text{QX})$ coming from IMRT treatments. Assuming that no prescription dose escalation is made and the average number of patients treated per day is unchanged, $W_{\text{dir}}(\text{QX})$ will remain the same with or without IMRT delivery. The following relationships then apply:

$$W_{\text{dir}}(\text{QX})=W_{\text{sca}}(\text{QX})⩽W_{L}(\text{QX})=(1-r+r*C)*W_{\text{dir}}(\text{QX})$$

and

$$U_{\text{dir}}(\text{QX},G_{k})=U_{\text{sca}}(\text{QX},G_{k})\approx U_{L}(\text{QX},G_{k}).$$

For these conditions only one set of use factors is needed and the leakage workload needs special attention. Two particular situations are noted: (1) When r=0 (no IMRT) the workload and use factors are the same for all three sources. (2) When r=1 (for a dedicated IMRT accelerator) $W_{L}(\text{QX})=C^{*}W_{\text{dir}}(\text{QX})$, but the use factor distributions remain roughly the same. The additional secondary barrier leakage TVL's required because of IMRT is shown in Table I for various *r* and *C* combinations.

**Table I:** The additional leakage TVL's needed for leakage contribution at secondary barriers. r is the percent of clinical treatments that are IMRT and C is the average MU to cGy ratio for the IMRT technique employed.

*r*	*C*	$W_{L}$	Additional TVL's
0%		$1.0W_{\text{dir}}$	0 TVL
50%	4	$2.5W_{\text{dir}}$	0.40 TVL
50%	10	$5.5W_{\text{dir}}$	0.74 TVL
100%	4	$4W_{\text{dir}}$	0.60 TVL
100%	10	$10W_{\text{dir}}$	1.0 TVL

(b) Next, consider clinical procedures consisting of “standard” and TBI treatments and no IMRT. A quantitative example will illustrate the situation. Assume $W_{\text{standard}}(\text{QX})=40\times 10^{3}$ cGy/wk and one TBI patient per week at 1200 cGy total dose. If the TBI treatment distance is 5 m from the x‐ray target, then $W_{\text{dir}}(\text{QX})=70\times 10^{3}\;\text{cGy}/\text{wk}=W_{L}(\text{QX})$, whereas $W_{\text{sca}}(\text{QX})=40\times 10^{3}$ cGy/wk. Similarly, the use factor distributions for direct, leakage, and scatter are substantially altered by the TBI contribution. Simplistically, if the {left, right, up, down} use factor distribution for direct, leakage, and scatter without TBI is {1/4, 1/4, 1/4, 1/4}, the addition of TBI, in this example, would yield new use factor distributions of {4/7, 1/7, 1/7, 1/7} for the direct and leakage sources while the scatter distribution remains unchanged.

(c) Finally, we consider the situation when both TBI and IMRT are performed. When TBI's at a frequency of one or more per week are performed along with a fraction *r* of the patients being treated with IMRT we find

$$W_{\text{sca}}(\text{QX})<W_{\text{dir}}(\text{QX})<W_{L}(\text{QX})$$

and $U_{\text{sca}}(\text{QX},G_{k})$ is influenced by standard and IMRT treatments.

$U_{\text{dir}}$ and $U_{L}$ are influenced (differently) by standard, IMRT, and TBI treatment workloads. The shielding calculation procedure, while more complex, is the same: (a) Determine primary barrier requirements from the direct source workload and use factors; (b) Separately compute secondary barrier thickness needed for leakage and for scatter sources and then determine the combined thickness required.

## CONCLUSION

The determination of separate workloads for direct, leakage, and scatter radiations provides a means of extending the older NCRP methods for barrier calculation to be applicable when clinical treatment procedures like IMRT and TBI are introduced to the radiation oncology clinic. If the TBI contribution to the workloads is negligible, the scatter workload and direct workloads are equal. When IMRT procedures are introduced to an accelerator vault not previously designed for IMRT, it may be necessary to increase shielding on secondary barriers depending on the IMRT technique and percentage of patients so treated. New vault designs should increase secondary barriers if IMRT or TBI are anticipated. The admixture of IMRT, SRS, and TBI influences use factor distributions as well.

## ACKNOWLEDGMENTS

This analysis was performed in conjunction with activities of the NCRP Subcommittee SC‐46‐13 (on revision of NCRP Report 49 for shielding of therapy rooms) and AAPM Task Group 57 (accelerator vault shielding). The author is grateful for the assistance and encouragement committee members.
